# Sample size requirements to estimate key design parameters from external pilot randomised controlled trials: a simulation study

**DOI:** 10.1186/1745-6215-15-264

**Published:** 2014-07-03

**Authors:** M Dawn Teare, Munyaradzi Dimairo, Neil Shephard, Alex Hayman, Amy Whitehead, Stephen J Walters

**Affiliations:** 1 Design, Trials and Statistics Group, School of Health and Related Research, University of Sheffield, Regent Court, 30 Regent Street, S1 4DA Sheffield, UK

**Keywords:** sample size, feasibility studies, pilot studies, binary outcomes, continuous outcomes, RCTs

## Abstract

**Background:**

External pilot or feasibility studies can be used to estimate key unknown parameters to inform the design of the definitive randomised controlled trial (RCT). However, there is little consensus on how large pilot studies need to be, and some suggest inflating estimates to adjust for the lack of precision when planning the definitive RCT.

**Methods:**

We use a simulation approach to illustrate the sampling distribution of the standard deviation for continuous outcomes and the event rate for binary outcomes. We present the impact of increasing the pilot sample size on the precision and bias of these estimates, and predicted power under three realistic scenarios. We also illustrate the consequences of using a confidence interval argument to inflate estimates so the required power is achieved with a pre-specified level of confidence. We limit our attention to external pilot and feasibility studies prior to a two-parallel-balanced-group superiority RCT.

**Results:**

For normally distributed outcomes, the relative gain in precision of the pooled standard deviation (SD_*p*_) is less than 10% (for each five subjects added per group) once the total sample size is 70. For true proportions between 0.1 and 0.5, we find the gain in precision for each five subjects added to the pilot sample is less than 5% once the sample size is 60. Adjusting the required sample sizes for the imprecision in the pilot study estimates can result in excessively large definitive RCTs and also requires a pilot sample size of 60 to 90 for the true effect sizes considered here.

**Conclusions:**

We recommend that an external pilot study has at least 70 measured subjects (35 per group) when estimating the SD_*p*_ for a continuous outcome. If the event rate in an intervention group needs to be estimated by the pilot then a total of 60 to 100 subjects is required. Hence if the primary outcome is binary a total of at least 120 subjects (60 in each group) may be required in the pilot trial. It is very much more efficient to use a larger pilot study, than to guard against the lack of precision by using inflated estimates.

## Background

In 2012/13, the National Institute for Health Research (NIHR) funded £208.9 million of research grants across a broad range of programmes and initiatives to ensure that patients and the public benefit from the most cost-effective up-to-date health interventions and treatments as quickly as possible [1]. A substantial proportion of these research grants were randomised controlled trials (RCTs) to assess the clinical effectiveness and cost-effectiveness of new health technologies. Well-designed RCTs are widely regarded as the least biased research design for evaluating new health technologies and decision-makers, such as the National Institute for Health and Care Excellence (NICE), are increasingly looking to the results of RCTs to guide practice and policy.

RCTs aim to provide precise estimates of treatment effects and therefore need to be well designed to have good power to answer specific clinically important questions. Both overpowered and underpowered trials are undesirable and each poses different ethical, statistical and practical problems. Good trial design requires the magnitude of the clinically important effect size to be stated in advance. However, some knowledge of the population variation of the outcome or the event rate in the control group is necessary before a robust sample size calculation can be done. If the outcome is well established, these key population or control parameters can be estimated from previous studies (RCTs or cohort studies) or through meta-analyses. However, in some cases finding robust estimates can pose quite a challenge if reliable data, for the proposed trial population under investigation, do not already exist.

A systematic review of published RCTs with continuous outcomes found evidence that the population variation was underestimated (in 80% of reported endpoints) in the sample size calculations compared to the variation observed when the trial was completed [2]. This study also found that 25% of studies were vastly underpowered and would have needed five times the sample size if the variation observed in the trial had been used in the sample size calculation. A more recent review of trials with both binary and continuous outcomes [3] found that there was a 50% chance of underestimating key parameters. However, they too found large differences between the estimates used in the sample size calculation compared to the estimates derived from the definitive trial. This suggests that many RCTs are indeed substantially underpowered or overpowered. A systematic review of RCT proposals reaching research ethics committees [4] found more than half of the studies included did not report the basis for the assumed values of the population parameters. So the values assumed for the key population parameters may be the weakest part of the RCT design.

A frequently reported problem with publicly funded RCTs is that the recruitment of participants is often slower or more difficult than expected, with many trials failing to reach their planned sample size within the originally envisaged trial timescale and trial-funding envelope. A review of a cohort of 122 trials funded by the United Kingdom (UK) Medical Research Council and the NIHR Health Technology Assessment programme found that less than a third (31%) of the trials achieved their original patient recruitment target, 55/122 (45.1%) achieved less than 80% of their original target and half (53%) were awarded an extension [5]. Similar findings were reported in a recently updated review [6]. Thus, many trials appear to have unrealistic recruitment rates. Trials that do not recruit to the target sample size within the time frame allowed will have reduced power to detect the pre-specified target effect size.

Thus the success of definitive RCTs is mainly dependent on the availability of robust information to inform the design. A well-designed, conducted and analysed pilot or feasibility trial can help inform the design of the definitive trial and increase the likelihood of the definitive trial achieving its aims and objectives. There is some confusion about terminology and what is a feasibility study and what is a pilot study. UK public funding bodies within the NIHR portfolio have agreed definitions for pilot and feasibility studies [7]. Other authors have argued against the use of the term ‘feasibility’ and distinguish three types of preclinical trial work [8].

### Distinguishing features of pilot and feasibility studies

NIHR guidance states:

*Feasibility studies are pieces of research done before a main study in order to answer the question ‘Can this study be done?’. In this context they can be used to estimate important parameters that are needed to design the main study*[9]*. For instance:*

i) *standard deviation of the outcome measure, which is needed in some cases to estimate sample size;*

ii) *willingness of participants to be randomised;*

iii) *willingness of clinicians to recruit participants;*

iv) *number of eligible patients over a specific time frame;*

v) *characteristics of the proposed outcome measure and in some cases feasibility studies might involve designing a suitable outcome measure;*

vi) *follow-up rates, response rates to questionnaires, adherence/compliance rates, intracluster correlation coefficients in cluster trials, etc.*

Feasibility studies for randomised controlled trials may themselves not be randomised. Crucially, feasibility studies do not evaluate the outcome of interest; that is left to the main study.

If a feasibility study is a small RCT, it need not have a primary outcome and the usual sort of power calculation is not normally undertaken. Instead the sample size should be adequate to estimate the critical parameters (e.g. recruitment rate) to the necessary degree of precision.

*Pilot trials are a version of the main study that is run in miniature to test whether the components of the main study can all work together*[9]*. It will therefore resemble the main study in many respects, including an assessment of the primary outcome. In some cases this will be the first phase of the substantive study and data from the pilot phase may contribute to the final analysis; referred to as an internal pilot. Or at the end of the pilot study the data may be analysed and set aside, a so-called external pilot*[10]*.*

For the purposes of this paper we will use the term pilot study to refer to the pilot work conducted to estimate key parameters for the design of the definitive trial. There is extensive but separate literature on two-stage RCT designs using an internal pilot study [11-14].

There is disagreement over what sample size should be used for pilot trials to inform the design of definitive RCTs [15-18]. Some recommendations have been developed although there is no consensus on the matter. Furthermore, the majority of the recommendations focus on estimating the variability of a continuous outcome and relatively little attention is paid to binary outcomes. The disagreement stems from two competing pressures. Small studies can be imprecise and biased (as defined here by comparing the median of the sampling distribution to the true population value), so larger sample sizes are required to reduce both the magnitude of the bias and the imprecision. However, in general participants measured in an external pilot or feasibility trial do not contribute to the estimation of the treatment effect in the final trial, so our aim should be to maintain adequate power while keeping the total number of subjects studied to a minimum. Recently some authors have promoted the practice of taking account of the imprecision in the estimate of the variance for a continuous outcome. Several suggest the use of a one-sided confidence interval approach to guarantee that power is at least what is required more than 50% of the time [15,18,19].

This paper aims to provide recommendations and guidelines with respect to two considerations. Firstly, what is the number of subjects required in an external pilot RCT to estimate the uncertain critical parameters (SD for continuous outcomes; and consent rates, event rates and attrition rates for binary outcomes) needed to inform the design of the definitive RCT with a reasonable degree of precision? Secondly, how should these estimates from the pilot study be used to inform the sample size (and design) for the definitive RCT? We shall assume that the pilot study (and the definitive RCT) is a two-parallel-balanced-group superiority trial of a new treatment versus control.

For the purposes of this work we assume that the sample size of the definitive RCT is calculated using a level of significance and power argument. This is the approach that is currently commonly employed in RCTs; however, alternative methods to calculate sample size have been proposed, such as using the width of confidence intervals [20] and Bayesian approaches to allow for uncertainty [21-23].

## Methods

Our aim is to demonstrate the variation in estimates of population parameters taken from small studies. Though the sampling distributions of these parameters are well understood from statistical theory, we have chosen to present the behaviours of the distributions through simulation rather than through the theoretical arguments as the visual representation of the resulting distributions makes the results accessible to a wider audience.

Randomisation is not a necessary condition for estimating all parameters of interest. However, it should be noted that some parameters of interest during the feasibility phase are related to the randomisation procedure itself, such as the rate of willingness to be randomised, and the rate of retention or dropout in each randomised arm. In addition, randomisation ensures the equal distribution of known and unknown covariates on average across the randomised groups. This ensures that we can estimate parameters within arms without the need to worry about confounding factors. In this work we therefore decided to allow for the randomisation of participants to mimic the general setting for estimating all parameters, although it is acknowledged that some parameters are independent of randomisation.

We first consider a normally distributed outcome measured in two groups of equal size. We considered study groups of from 10 to 80 subjects using increments of five per group. For each pilot study size, 10,000 simulations were performed. Without loss of generality, we assumed the true population mean of the outcome is 0 and the true population variance is 1 (and that these are the same in the intervention and control groups). We then use the estimate of the SD, along with other information, such as the minimum clinically important difference in outcomes between groups, and Type I and Type II errors levels, to calculate the required sample size (using the significance thresholds approach) for the definitive RCT.

The target difference or effect size that is regarded as the minimum clinically important difference is usually the difference in the means when comparing continuous outcomes for the intervention with those of the control group. This difference is then converted to a standardised effect size by dividing by the population SD. More details of the statistical hypothesis testing framework in RCTs can be found in the literature [24,25].

For a two-group pilot RCT we can use the SD estimate from the new treatment group or the control/usual care group or combine the two SD estimates from the two groups and use a pooled standard deviation (SD_*p*_) estimated from the two-group specific sample SDs. For sample size calculations, we generally assume the variability of the outcome is the same or equal in both groups, although this assumption can be relaxed and methods are available for calculating sample sizes assuming unequal SDs in each group [26,27]. This is analogous to using the standard *t*-test with two independent samples (or multiple linear regression), which assumes equal variances, to analyse the outcome data compared with using versions of the *t*-test that do not assume equal variances (e.g. Satterthwaite’s or Welch’s correction).

We assume binary outcomes are binomially distributed and consider a number of different true population proportions as the variation of proportion estimator is a function of the true proportion. When estimating an event rate, it may not always be appropriate to pool the two arms of the study so we study the impact of estimating a proportion from a single arm where the study size increases in steps of five subjects. We considered true proportions in the range 0.1 to 0.5 in increments of 0.05. For each scenario and sample size, we simulated the feasibility study at least 10,000 times depending on the assumed true proportion. For the binary outcomes, the number of simulations was determined by requiring the proportion to be estimated within a standard error of 0.001. Hence, the largest number of simulations required was 250,000 when the true proportion was equal to 0.5. Simulations were performed in Stata version 12.1 [28] and R version 13.2 [29].

### Normally distributed outcomes

For each simulation, sample variances were calculated for each group ($s_{1}^{2}$ and $s_{2}^{2}$) and the pooled SD was calculated as follows:

(1) $${\mathrm{SD}}_{p}=\sqrt{\left(\frac{\left(s_{1}^{2}+s_{2}^{2}\right)}{2}\right)}.$$

We also computed the standard error of the sample pooled SD which is

(2) $$\mathrm{se}\left({\mathrm{SD}}_{p}\right)=\frac{{\mathrm{SD}}_{p}}{\sqrt{2\left(n-1\right)}}\cdot$$

To quantify the relative change in precision, we compared the average width of the 95% confidence intervals (WCI_2*n*_) for the SD_*p*_ for study sizes of 2*n* with the average width when the study size was increased to 2(*n* + 5). We use the width of the confidence interval as this provides a measure of the precision of the estimate.

Given the sampling distribution of the SD, its lower and upper 95% confidence limits are given by:

(3) $$\left(\sqrt{\frac{2\left(n-1\right)}{\chi_{0.025,2\left(n-1\right)}}}{\mathrm{SD}}_{p}\;\mathrm{and}\;\sqrt{\frac{2\left(n-1\right)}{\chi_{0.975,2\left(n-1\right)}}}{\mathrm{SD}}_{p}\right),\;$$

and the relative percentage gain in precision is quantified as the reduction in 95% confidence interval width if the sample size is increased by five per group:

(4) $$\left(\frac{\mathrm{WC}I_{2n}-\;\mathrm{WC}I_{2\left(n+5\right)}}{\mathrm{WC}I_{2n}}\right)\;\times \;100.$$

Bias is assessed by subtracting the true value from each estimate and taking the mean of these differences.

We also consider the impact of adjusting the SD estimate from the pilot as suggested originally by Browne in 1995 [15]. Here a one-sided confidence limit is proposed to give a corrected value. If we used the 50% one-sided confidence limit, this would adjust for the bias in the estimate, and this correction has also been proposed when using small pilots [17]. If we specify 50% confidence then our power will be as required 50% of the time. Sim and Lewis [18] suggest that it is reasonable to require that the sample size calculation guarantees the desired power with a specified level of confidence greater than 50%. For the sake of illustration, we will consider an 80% confidence level for the inflation factor. So we require the confidence interval limit associated with 80% confidence above that value. Hence the inflation factor to apply to the SD_*p*_ from the pilot is:

(5) $$\sqrt{\frac{2\left(n-1\right)}{\chi_{0.8,2\left(n-1\right)}}\cdot }$$

To consider the impact on power and planned sample size, we need to state reasonable specific alternative hypotheses. In trials, it is uncommon to see large differences between treatments so we considered small to medium standardised effect sizes (differences between the group means) of 0.2, 0.35 and 0.5 [30]. For each true effect size of 0.2, 0.35 or 0.5, we divide by the SD_*p*_ estimate for each replicate, and use this value to calculate the required sample size. For each simulated pilot study, we calculate the planned sample size for the RCT assuming either the unadjusted or adjusted SD_*p*_ estimated from the pilot. Using this planned sample size (where the SD_*p*_ has been estimated) we then calculate the true power of the planned study assuming that we know that the true population SD_*p*_ is in fact 1.

### Binary outcomes

We consider that the binary outcome will be measured for one homogeneous group only. The following is repeated for each true population success probability. We examined nine true success probabilities from 0.1 to 0.5 in intervals of 0.05. We considered 41 different pilot study sizes ranging from 10 to 200 consisting of multiples of five subjects. The subscripts *i* and *j* are used to denote the true proportion and the pilot study size, respectively. For each simulated pilot study of size *n*_*j*_, the number of successes  (*Y*_*ij*_ ~ Bin(*n*_*j*_, *θ*_*i*_)) in the simulation *n*_*j*_ are counted. First, the observed proportions, $\;{\hat{\theta }}_{i}$, for each of the nine true success probabilities were calculated by:

(6) $${\hat{\theta }}_{i}=\;\frac{Y_{\mathrm{ij}}}{n_{j}}.$$

The associated 95% confidence interval was calculated using Wilson’s score [21] given by:

(7) $$\frac{\left({\hat{\theta }}_{i}+\frac{z_{\alpha /2}^{2}}{2n_{j}}\;\pm \;z_{\alpha /2}\;\sqrt{\frac{{\hat{\theta }}_{i}\left(1-{\hat{\theta }}_{i}\right)+\frac{z_{\alpha /2}^{2}}{4n_{j}}}{n_{j}}}\;\right)}{\left(1+\frac{z_{\alpha /2}^{2}}{n_{j}}\right)}$$

Second, this process was repeated for *N*_*s*_ (the number of simulations needed to estimate the true success probability to within 0.1% of its standard error) and the average observed success probability for each of the nine true success probabilities (*θ*) for a given fixed pilot size were calculated as follows:

(8) $${\bar{\theta }}_{i}=\frac{1}{N_{s}}\sum_{k=1}^{N_{s}}{\hat{\theta }}_{\mathrm{ik}},$$

where ${\hat{\theta }}_{\mathrm{ik}}$ is ${\hat{\theta }}_{i}$ for the *k*th simulated pilot study. Third, due to the relatively small sample size of the pilot trials, we computed the mean width of the 95% confidence interval of the true success probability averaged over *N*_*s*_ simulations using the Wilson’s score method [31] for a fixed sample size, which is given by:

(9) $$\frac{1}{N_{s}}\sum_{k=1}^{N_{s}}\frac{\left(2z_{a/2}\;\sqrt{\frac{{\hat{\theta }}_{\mathrm{ik}}\left(1-{\hat{\theta }}_{\mathrm{ik}}\right)\;+\frac{z_{a/2}^{2}}{4n_{j}}}{n_{j}}}\right)}{\left(1+\frac{z_{a/2}^{2}}{n_{j}}\right)}\;.$$

The relative percentage gain in precision around the true binomial proportion per increase of five study participants is defined as before:

(10) $$\left(\frac{\mathrm{WC}I_{n_{j}}-\;\mathrm{WC}I_{n_{j}+5}}{\mathrm{WC}I_{{}_{n_{j}}}}\right)\;\times \;100.$$

As for the continuous outcomes, bias is assessed by subtracting the true population value from each estimate and taking the signed mean of these. We also report the 95% coverage probability [32].

## Results and discussion

### Normally distributed outcomes

**Figure 1:**
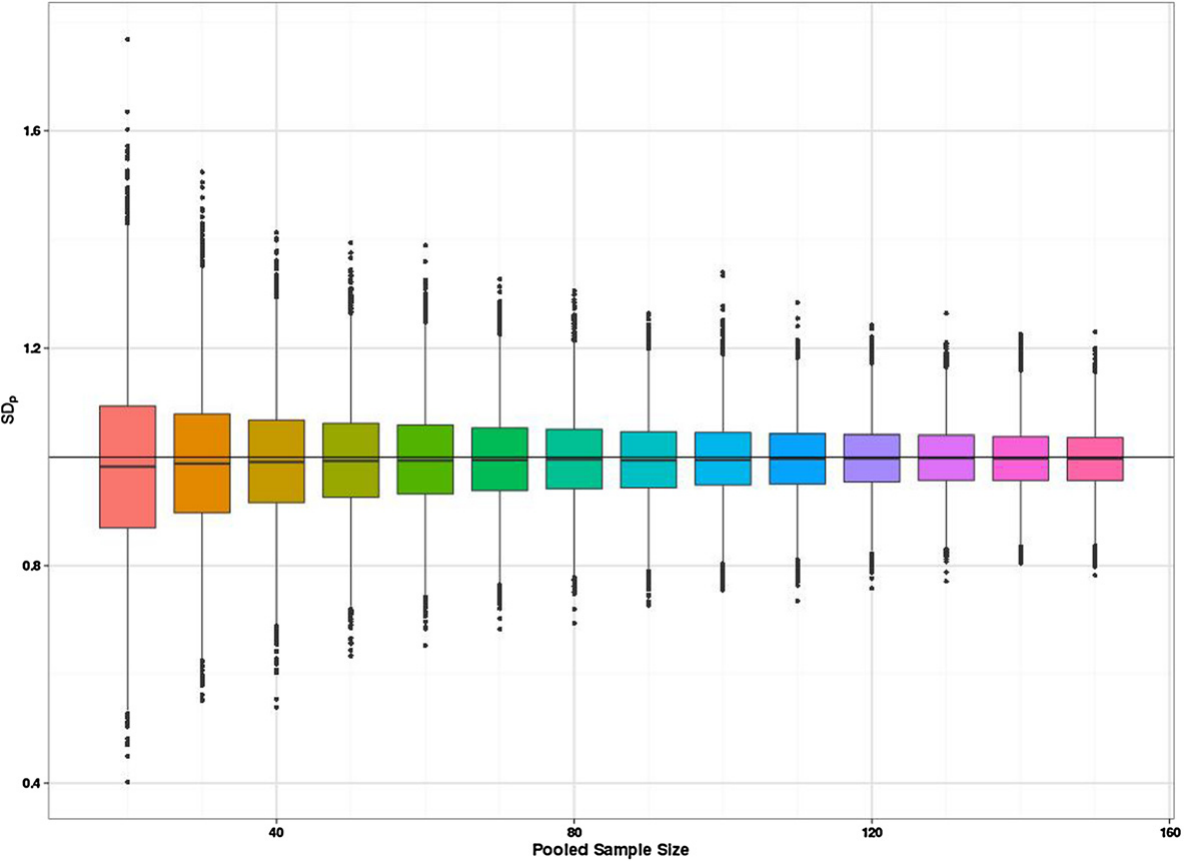
**Multiple box and whisker plot of SD**_***p ***_**estimates by pooled sample size of the pilot study.** The vertical axis shows the value of the SD_*p*_ estimate for 10,000 simulations per pilot study size. The horizontal axis is graduated by the pooled pilot study size.

Figure 1 is a multiple box and whisker plot of the resulting distributions of the sample SD_*p*_. Under our simulations the true SD is equal to 1. Figure 1 clearly shows that the spread of the estimates reduces as the pooled sample size increases and the distribution of the estimated SD_*p*_ also becomes more symmetric as the pooled sample size increases. So the bias and skew is more marked for smaller sample sizes. The direction of the bias means that the SD tends to be underestimated. Once the total sample size is above 50 the average bias becomes negligible and is less than 0.005 below the true value. However, what is more noticeable is the large variation in the sampling distribution for the smaller sample sizes and considerable sampling variation remains even with a large sample size.

**Figure 2:**
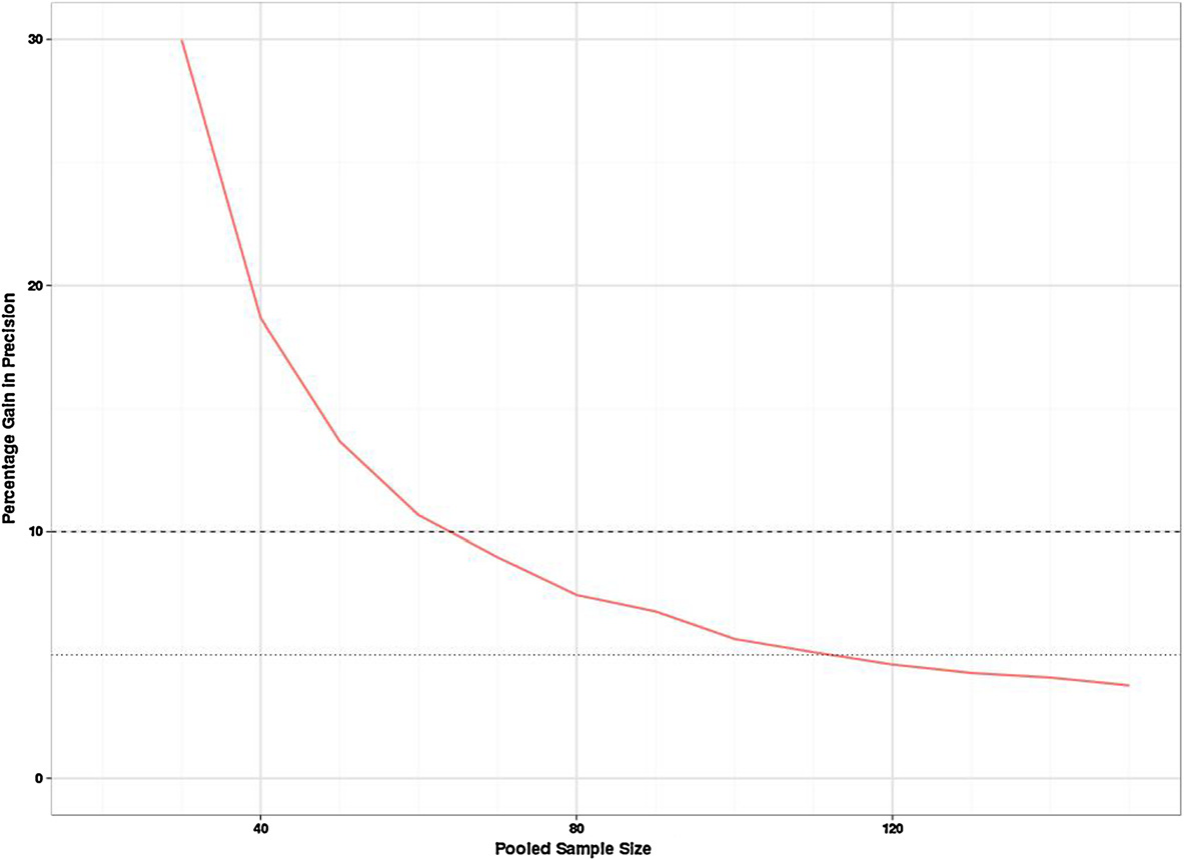
**Percentage gain in precision of SD**_***p ***_**on increasing the pooled sample size.** This shows the relative reduction in the average width of the confidence interval when an additional five subjects are added to a group.

**Figure 3:**
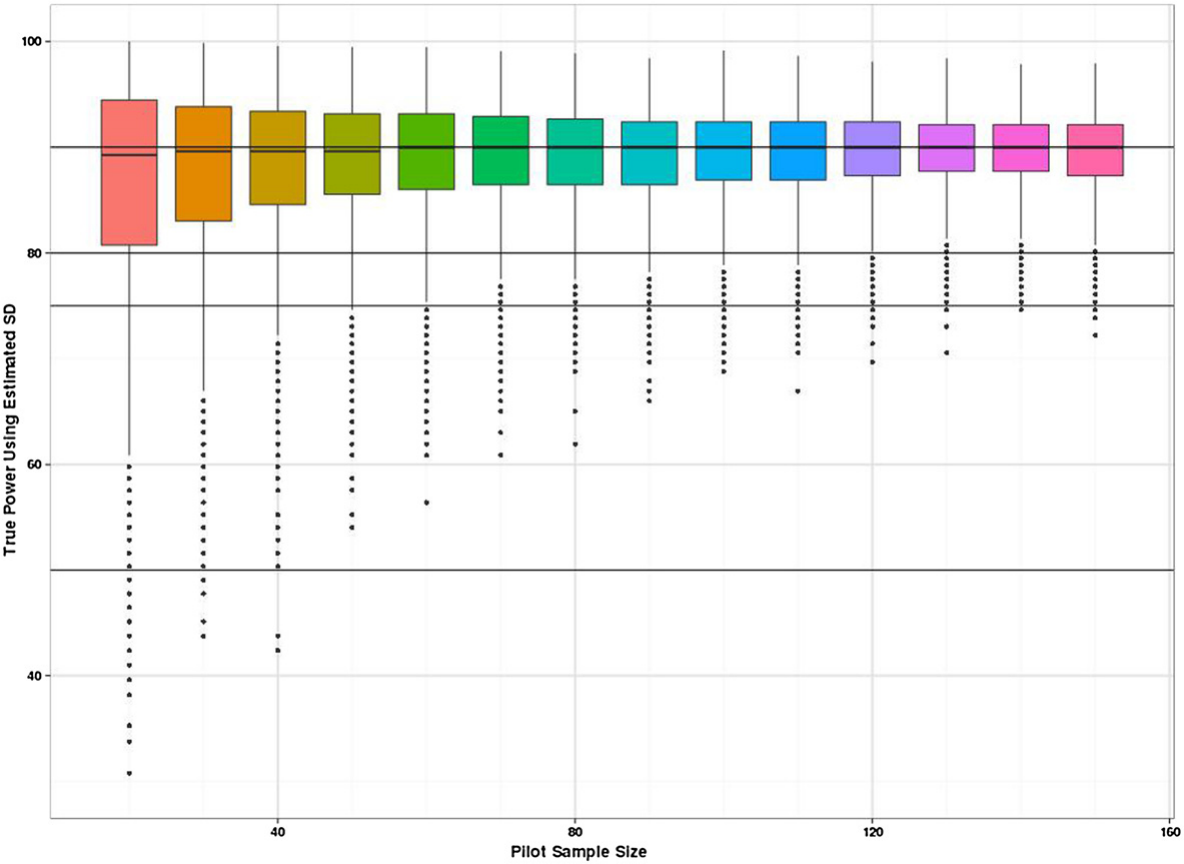
**Distribution of planned RCT study power when using the SD**_***p***_**estimate derived from the pilot study.** The planned study size is used to calculate the true power if SD = 1 is assumed. The graph shown is for a true effect size of 0.2. The vertical axis is true power. The *x*-axis shows the size of the two-arm pilot study.

**Figure 4:**
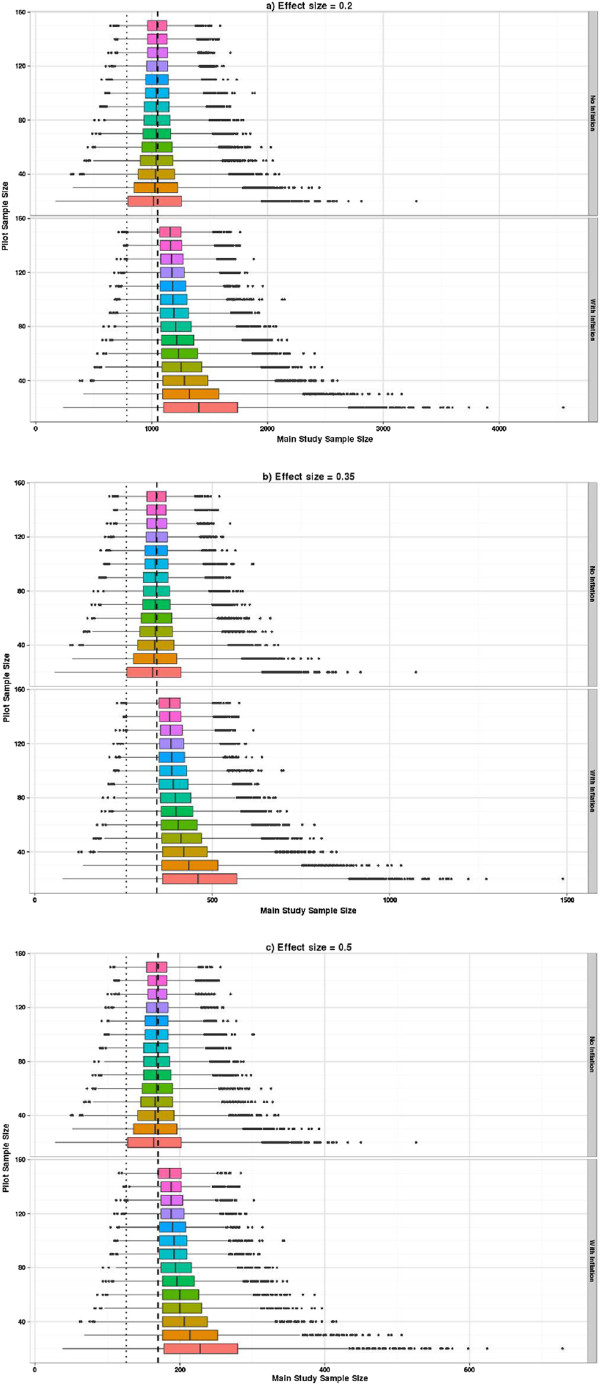
**Distribution of planned sample sizes using crude SD*p *estimates and adjusting for a specified level of confidence. (a)** Effect size = 0.2. **(b)** Effect size = 0.35. **(c)** Effect size = 0.5. The upper part of each graph shows the distribution of planned sample sizes by pilot study size. The lower part shows the same but using the inflation adjustment to guarantee the specified power with 80% confidence. The *x*-axis shows the planned sample size and the vertical axis shows the pilot study size. The dashed vertical line shows the sample size associated with a true power of 90% and the dotted line for 80%.

Figure 2 shows the percentage gain in precision (the width of the confidence interval for the SD_*p*_) when adding ten more participants to the sample (five to each group). Precision increases with sample size, however, the relative gain in precision (while always positive) decreases with increasing sample size. With a total sample size of 70, there is a less than 10% gain in precision when adding further participants to the study size. So in terms of good precision and minimal bias (for a continuous outcome) a total sample size of 70 seems desirable for a pilot study.Figure 3 shows the distribution of true power for the planned sample sizes for the specific alternative effect size of 0.2, assuming we require 90% power at the 5% two-sided significance level. The true power distribution for the other effects sizes is very similar (it can be shown that conditional on the estimated SD from the pilot, the distributions should be the same but rounding up to integers causes slight changes at small sample sizes). As anticipated, this figure shows a large variation in power for the smaller sample sizes. However, even with the relatively small pilot sample size of 20, the planned studies do have at least 80% power to detect the target effect size (when we have stated we desire 90% power) more than 75% of the time. Figure 3 also shows that the true power frequently exceeds 90% but the cost of this higher power in terms of total participants cannot be quantified from this figure. By contrast Figure 4 is able to show the ‘cost’ of the higher power translated into the sample size scale.

**Figure 5:**
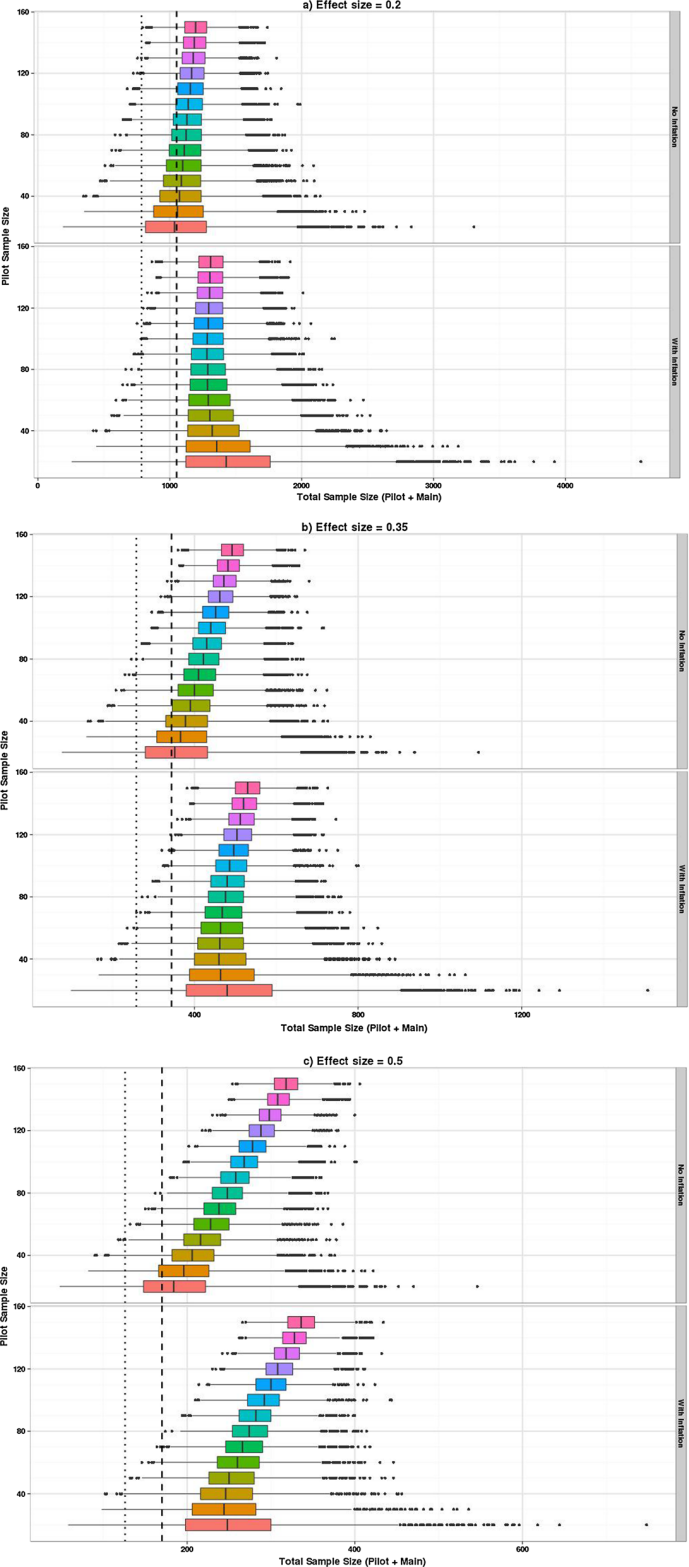
**Distribution of total sample size required when using pilot sample derived SD*p *estimated with and without inflation. (a)** Effect size = 0.2. **(b)** Effect size = 0.35. **(c)** Effect size = 0.5. This figure is similar to Figure 4; however, now the total sample size includes the pilot study size. The dashed and dotted vertical lines represent the sample size required for 90% and 80% power, respectively, if the true SD were known and the pilot study were not necessary.

Figure 4 shows the distribution of the planned sample size when using the estimated SD_*p*_ from the pilot (with and without inflation of the SD_*p*_). It can be seen that the overall shape of these plots is similar for all three effects sizes, but the planned sample sizes are proportionately higher as the effect size reduces. Figure 4a shows the sample size (for a true difference between the means of 0.2) using the unadjusted SD_*p*_ (upper plot) and the inflated SD_*p*_ (lower plot). Using the inflated SD_*p*_ means we have specified that we want our planned study to have 90% power with 80% confidence or certainty. By comparing these two plots and superimposing the sample size of 1,052, which is what we would actually need to detect an effect size of 0.2 with 90% power and 5% two-sided significance when the true SD is known to be equal to 1, you can readily see the effect of the inflation factor. Figures 4b,c present the same contrasts as Figure 4a but for a true difference between the means of 0.35 and 0.5, respectively. The main impact of the inflation factor is to guarantee that 80% of the planned studies are in fact *larger* than they need to be, and for the smaller pilots this can be up to 50% larger than necessary. If only the unadjusted crude estimates from the pilot are used to plan the future study, though we aim for at least 50% of studies to be powered at 90%, inspection of the percentiles shows that that the planned sample size delivers at least 80% power with 90% confidence, when a pilot study of at least 70 is used. Researchers need to consider carefully the minimum level of power they are prepared to tolerate for a worst-case scenario when the population variance is overestimated.Figure 5 adds the size of the pilot study to the planned study size so the distribution of the overall number of subjects required can be seen. The impact of the inflation factor now depends on the true effect size. If we are planning to use the inflation factor then when the effect size is 0.5 a pilot study of around 30 is optimal. However, the same average number of subjects would result using unadjusted estimates from a pilot study of size 70, and this would result in a smaller variation in planned study size. For the effect size of 0.2 then the optimal pilot study size if applying the inflation factor is around 90, but this optimal size still results in larger overall sample sizes than just using unadjusted estimates from pilot studies of size 150.

### Binary outcomes

**Figure 6:**
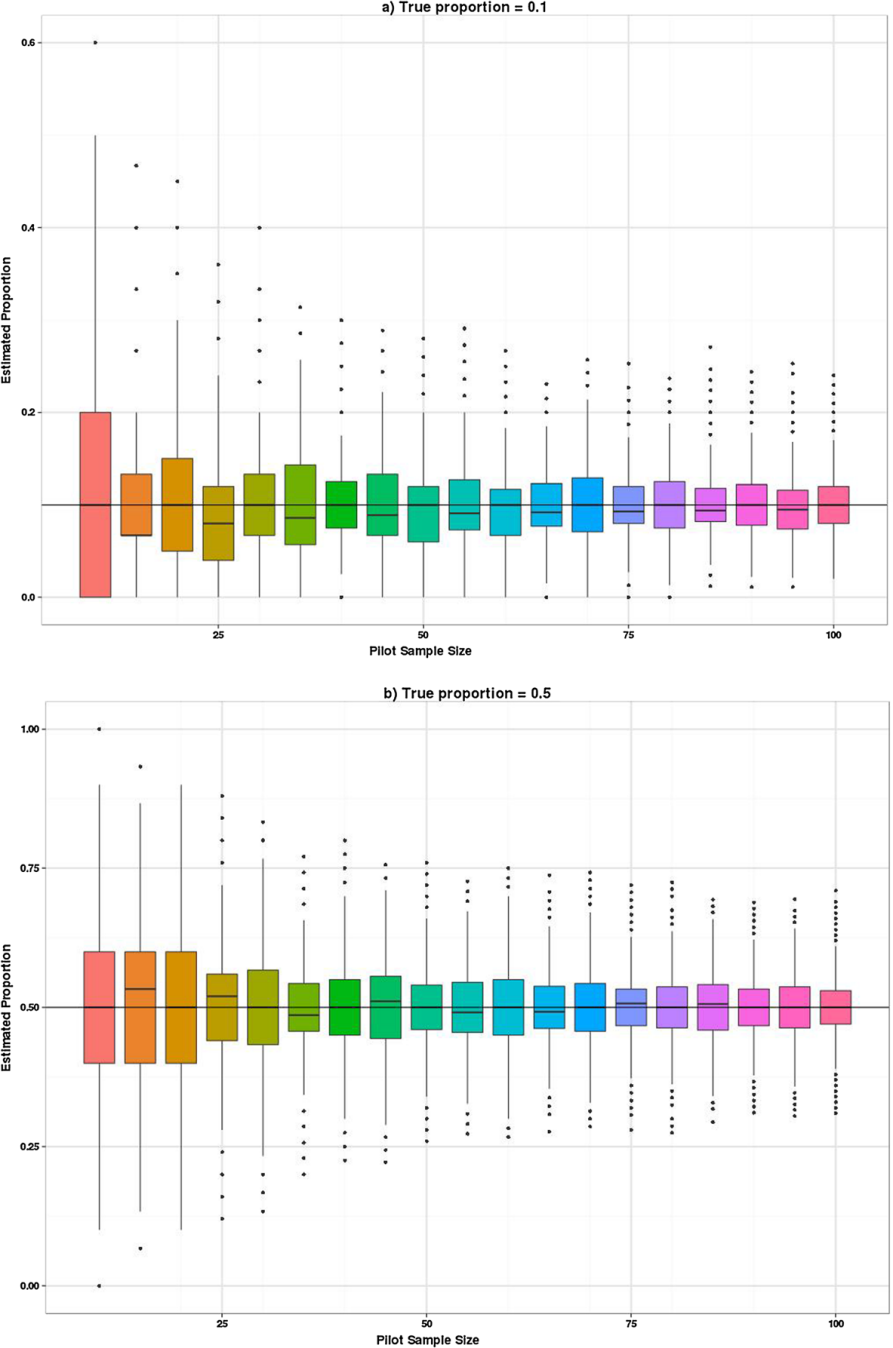
**Distribution of estimated event rates on increasing sample size.** Distributions for a true event rate of 0.1 **(a)** and a true event rate of 0.5 **(b)**.

**Figure 7:**
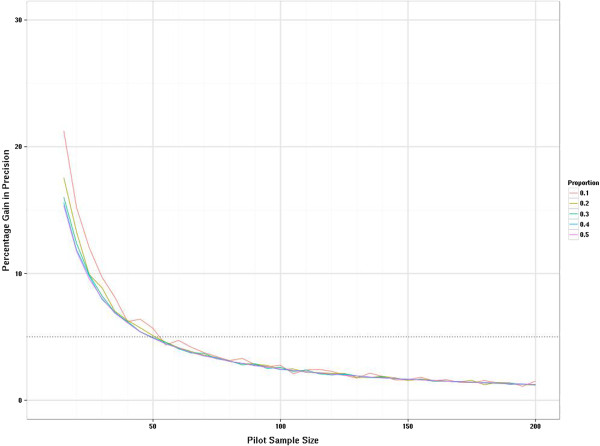
**Distribution of relative gain in precision for binary outcomes as pilot study size increases.** This graph compares the width of the confidence intervals for *n* + 5 subjects and *n* subjects. This is scaled by the width of the interval when there are *n* subjects.

**Figure 8:**
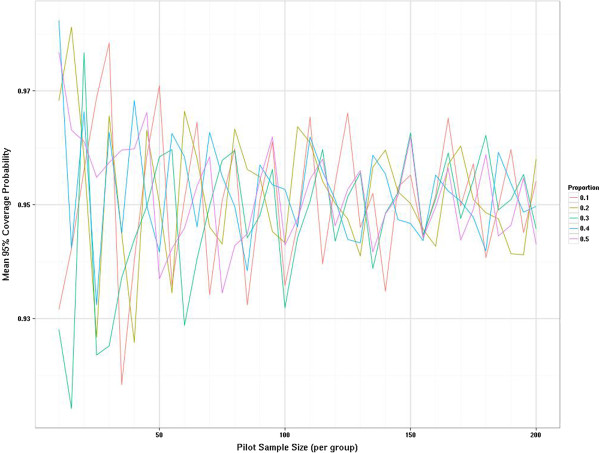
Distribution of mean coverage probability by true proportion and pilot sample size.

The sampling distribution when estimating a proportion is a function of the true population proportion so it seems unwise to estimate this from a pooled group unless it is a measure independent of treatment group and there is a strong assumption of equality between groups. We have explored the sampling distributions of the proportions in increments of five rather than ten as we allow the possibility that this may be estimated from one arm. As statistical theory predicts the sampling variation is largest when the true proportion is 0.5 and reduces as the true proportion becomes more different from 0.5, we show the results for the two most extreme proportions considered, i.e. 0.1 and 0.5 (Figure 6). When the true proportion is 0.1 the sampling distribution is slightly skewed with a tendency to underestimate the true value even when uneven pilot arm sizes are used. However, when the true proportion is 0.5 there is no systematic bias in under- or overestimating the parameters from the pilot. Most of the fluctuation is due to deriving estimates from a sample size where the true proportion is not a possible outcome (e.g., if the true proportion is 0.5 but the sample size is 25, then the closest you can observe to the true value is 12/25 or 13/25). Once the pilot sample size is 60 or more then these fluctuations settle down. The relative percentage gain in the precision of estimates is formally presented in Figure 7, where the average width of the 95% confidence intervals for the proportion are compared with the average confidence interval width if another five subjects were added to the sample. This relative percentage gain in precision is shown for true proportions 0.1 and 0.5. For the continuous outcomes we suggested a cut-off of 10% as a threshold. For the binary outcomes we use the 5% threshold as we are moving in steps of five rather than ten. The relative percentage gain in the precision graph crosses the 5% threshold when the sample size is 55 to 60 and crosses the 3% threshold when the sample size is 100. Figure 8 shows the coverage probability for five of the true proportions as sample size increases. This shows how frequently the 95% confidence interval contains the true value. This graph shows considerable fluctuations. Once the sample size is 100 there is very little perceptible improvement in the coverage probability for the true proportions considered here.

## Conclusions

Our simulated data visually demonstrate the large sampling variation that is the main weakness when estimating key parameters from small sample sizes. Small samples sizes do lead to biased estimates, but the bias is negligible compared to the sampling variation. When we examine the relative percentage gain in precision by adding more subjects to the sample, our data suggest that a total of at least 70 may be necessary for estimating the standard deviation of a normally distributed variable with good precision, and 60 to 100 subjects in a single group for estimating an event rate seems reasonable. Treatment-independent parameters may be estimated by pooling the two groups, so in many cases our recommended sample size will be the total sample size. On average when the definitive RCT is planned using an estimate from a pilot study there will be a tendency for the planned study to be underpowered. However, if the definitive RCT is planned for a continuous outcome requiring a power of 90% then the true power will be 80% with at least 76% assurance provided the estimates come from a pilot with at least 20 subjects. We considered three realistic effect sizes of 0.2, 0.35 and 0.5 of a standard deviation to evaluate the impact of adjusting for the anticipated uncertainty in the estimate from the pilot when calculating the sample size for the planned RCT as was recently suggested [18]. For all of the effect sizes considered, it is not efficient to use small pilots and apply the inflation adjustment, as this will result in larger sample sizes (pilot plus main study) in total. Further, we only considered sample sizes planned when requiring 90% power, and examine the conditional power assuming we know the true alternative. On average using imprecise estimates but requiring high power will result in acceptable power with much less ‘cost’ as measured by total sample size. Hence, it is actually more efficient to use a large external pilot study to reduce the variation around the target power for the definitive RCT.

The implication of using estimates of key parameters from small pilot studies is the risk of both over- and underpowered studies. While overpowered studies may not seem such an acute problem, they are potentially a costly mistake and may result in a study being judged as prohibitively large. This would seem to be an argument in favour of utilising internal pilot studies, but an internal pilot requires the key design features of the trial to be fixed, so any change in measurement of the treatment effect following an internal pilot will lead to analysis difficulties.

A major and well-documented problem with published trials is under recruitment, where there is a tendency to recruit fewer subjects than targeted. One reason for under recruitment may well be that event rates such as recruitment and willingness to be randomised cannot be accurately estimated from small pilots, and in fact increasing the pilot size to between 60 and 100 per group may give much more reliable data on the critical recruitment parameters.

In reality, when designing external pilot trials, there is a need to balance two competing issues: maximising the precision (of the critical parameters you wish to estimate) and minimising the size of the external pilot trial, which impacts on resources, time and costs. Thus there is a trade-off between the precision (of the estimates of the critical parameters) and size (number of subjects) of the pilot study. When designing external pilot trials, researchers need to understand that they are trading off the precision of the estimates against the total sample size of the definitive study when they decide to have an external pilot study with a small sample size.

## Abbreviations

NICE: National Institute for Health and Care Excellence; NIHR: National Institute for Health Research; RCT: randomised control trial; SD: standard deviation; UK: United Kingdom.

## Competing interests

The authors declare that they have no competing interests.

## Authors’ contributions

NS, MD and AH contributed to the conceptual design, performed the simulations and summary statistical analysis, and produced the graphical output. MDT contributed to the design of the project, and drafted and revised the manuscript. AW contributed to study design and drafted the literature review. SJW contributed to study design, and the first draft and revisions of the manuscript. All authors read and approved the final manuscript.
